# A Peek Inside the Machines of Bacterial Nucleotide Excision Repair

**DOI:** 10.3390/ijms22020952

**Published:** 2021-01-19

**Authors:** Thanyalak Kraithong, Silas Hartley, David Jeruzalmi, Danaya Pakotiprapha

**Affiliations:** 1 Doctor of Philosophy Program in Biochemistry (International Program), Faculty of Science, Mahidol University, Bangkok 10400, Thailand; kraithong.t@gmail.com; 2 Department of Biochemistry, Faculty of Science, Mahidol University, Bangkok 10400, Thailand; 3 Center for Excellence in Protein and Enzyme Technology, Faculty of Science, Mahidol University, Bangkok 10400, Thailand; 4 Department of Chemistry and Biochemistry, City College of New York, New York, NY 10031, USA; silashartley@gmail.com; 5 Doctor of Philosophy Programs in Biochemistry, Biology and Chemistry, The Graduate Center of the City University of New York, New York, NY 10016, USA

**Keywords:** nucleotide excision repair, NER, DNA repair, DNA damage recognition, UvrA, UvrB, UvrD, Mfd

## Abstract

Double stranded DNA (dsDNA), the repository of genetic information in bacteria, archaea and eukaryotes, exhibits a surprising instability in the intracellular environment; this fragility is exacerbated by exogenous agents, such as ultraviolet radiation. To protect themselves against the severe consequences of DNA damage, cells have evolved at least six distinct DNA repair pathways. Here, we review recent key findings of studies aimed at understanding one of these pathways: bacterial nucleotide excision repair (NER). This pathway operates in two modes: a global genome repair (GGR) pathway and a pathway that closely interfaces with transcription by RNA polymerase called transcription-coupled repair (TCR). Below, we discuss the architecture of key proteins in bacterial NER and recent biochemical, structural and single-molecule studies that shed light on the lesion recognition steps of both the GGR and the TCR sub-pathways. Although a great deal has been learned about both of these sub-pathways, several important questions, including damage discrimination, roles of ATP and the orchestration of protein binding and conformation switching, remain to be addressed.

## 1. Introduction

The genetic programs of organisms require robust mechanisms to maintain integrity of information carried in the base sequence of double stranded DNA (dsDNA). Surprisingly, the intrinsic stability of dsDNA in water is low and alone does not match the imperative to preserve its information-bearing sequence intact [1,2,3,4]. In addition, the structure of dsDNA is readily compromised by products of cellular processes, such as reactive oxygen species, which can introduce nearly two dozen characterized nucleobase lesions [5]; as well, exogenous agents, such as UV radiation and environmentally-derived agents can react with and disrupt the structure of dsDNA [6]. It is estimated that each mammalian cell absorbs tens of thousands of damaging events each day [2]; since bacterial DNA inhabits a similar chemical environment, the rates for bacterial cells are anticipated to be similar. Left unaddressed, constant decoration with lesions has the potential to disrupt replication and transcription, and, crucially, to corrupt information contained within dsDNA. To prevent this, bacterial and eukaryotic cells have evolved at least six distinct pathways to protect information in genomic DNA: direct reversal, base excision repair (BER), nucleotide excision repair (NER), mismatch repair (MMR), homologous recombination (HR) and nonhomologous end joining (NHEJ) [7]. Here, we review one of these pathways, bacterial NER; the other pathways have also been reviewed recently [6,8,9,10,11,12,13,14,15].

**Figure 1:**
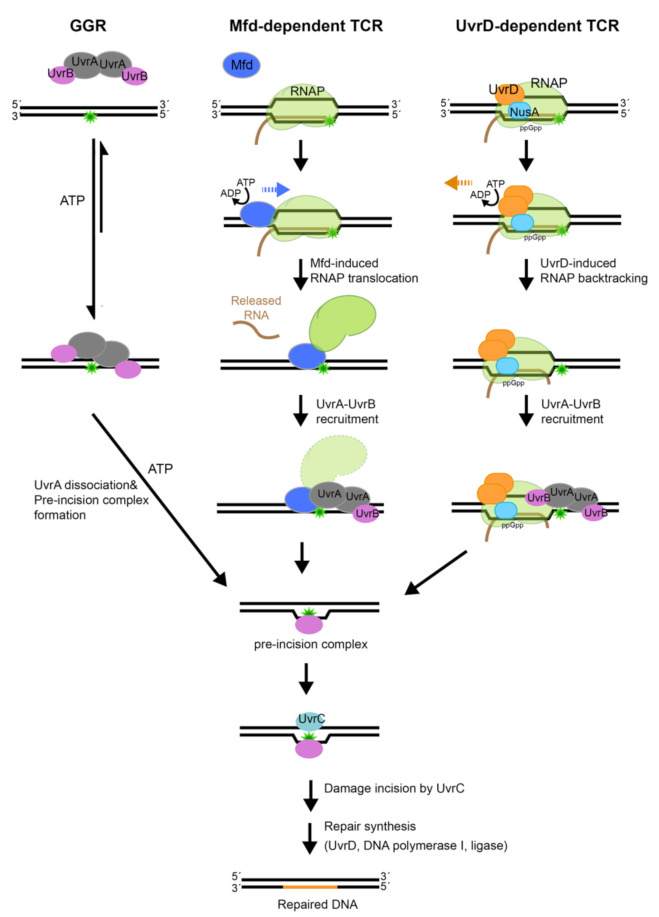
The nucleotide excision repair (NER) pathways. Nucleotide excision repair can occur via different sub-pathways. In global genome repair (GGR), the DNA damage is recognized by the UvrA_2_B_2_ damage sensor. Transcription couple repair (TCR), which removes lesions form the DNA strand that is being transcribed, is mediated by the Mfd protein. Mfd binds to RNA polymerase (RNAP) that is stalled by the lesion, pushes it forward to expose the lesion and recruits the nucleotide excision repair (NER) machinery. Recently, an alternative pathway has been proposed for TCR that is dependent on UvrD. In this newly proposed sub-pathway, UvrD binds to the stalled RNAP in the presence of the transcription elongation factor NusA and ppGpp and induces backtracking to expose the lesion; this is followed by lesion recognition by NER proteins. All three sub-pathways culminate in the formation of UvrB•DNA pre-incision complex, which commits the bound DNA for incision and subsequent repair.

NER operates in two modes (Figure 1): (1) global-genome repair (GGR) characterized by general patrol and repair of lesions within the genome and (2) transcription-coupled repair (TCR), a specialized NER process that interfaces with RNA polymerase to repair lesions on the transcribed strand.

During repair of damaged DNA, the two modes of NER, GGR and TCR, operate via several distinct stages: damage detection, damage verification, incision, damage excision, repair synthesis and ligation to restore the original DNA sequence; the main difference between the branches of NER centers on the initial detection of the lesion. In bacteria, the UvrA_2_ protein dimer, alone or in complex with the UvrB protein (UvrA_2_B_2_), uses the energy of ATP binding and hydrolysis to search the genome for lesions [16,17]. In the damage sensing complex, UvrA_2_ dimer alone contacts DNA [18], as it discriminates native from damaged DNA. Several hypotheses have been advanced to explain mechanisms that underlie damage sensing, however, a great deal remains to be understood [19,20,21,22]. Identification of a lesion and verification of the damage by UvrB trigger eviction of UvrA from the complex on DNA, with UvrB taking its place at the lesion to form the pre-incision complex [18]. The mechanisms associated with the handoff of damaged DNA from UvrA to UvrB, however, remain to be firmly established. UvrB subsequently recruits the UvrC endonuclease to form the incision complex. Within this complex, UvrC introduces two incisions. The first incision is made at the 4^th^ or 5^th^ phosphodiester bond on the 3′ side of the lesion; this is followed by the second incision at the 8^th^ phosphodiester bond on the 5′ side of the lesion [23]. Excision of the damage-containing ssDNA segment by the UvrD-helicase, repair synthesis by DNA polymerase I and ligation by DNA ligase [17] complete NER and lead to restoration of the original sequence.

The above sequence of steps can be found in both the GGR and TCR modes of NER, however, the latter mode includes several unique steps. Transcription-coupled repair is initiated when a lesion on the DNA strand that is being transcribed causes RNA polymerase to stall. In this scenario, the stalled RNA polymerase occludes the lesion, preventing its repair. In order to remove the DNA lesion and resume transcription, the stalled RNA polymerase needs to be displaced. To accomplish this, the Mfd (mutation frequency decline) transcription-repair coupling factor is recruited to the site [24]. Mfd removes the stalled RNA polymerase from DNA using ATP-dependent forward translocation [25] and recruits UvrA to the damaged DNA [26,27]. Recently, an alternative pathway for TCR has been proposed that leverages UvrD, NusA, together with guanosine 3′, 5′—bis(diphosphate) (ppGpp), to stimulate back-tracking of RNA polymerase [28,29]. Both branches of TCR culminate in the binding of UvrA and UvrB to damaged DNA, after which the sequence of steps as outlined above for GGR commences. Notably, although molecular mechanisms of NER are conserved from bacteria to humans, they are implemented by unrelated proteins [30].

## 2. The UvrA DNA Damage Sensor

**Figure 2:**
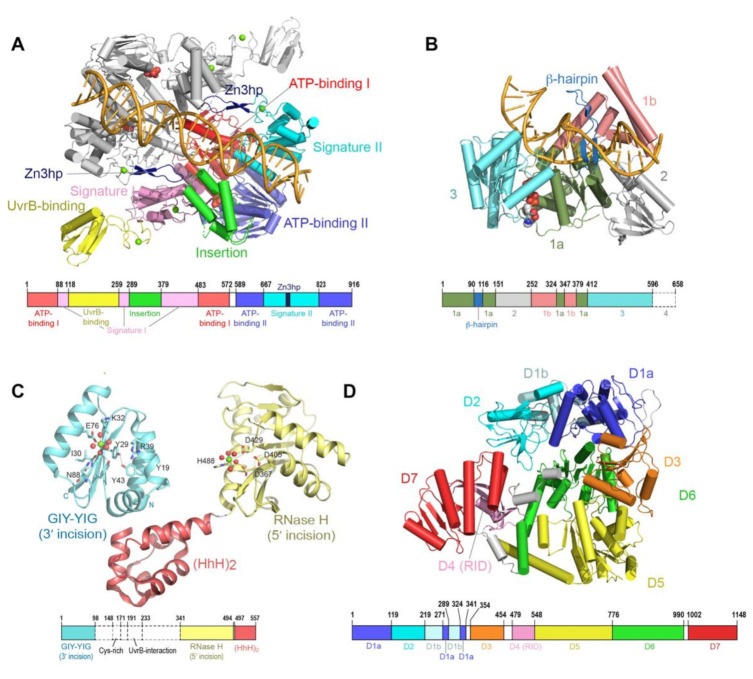
Architecture of nucleotide excision repair proteins. (**A**) Structure of *Thermotoga maritima (Tma)* UvrA dimer bound to lesion-containing duplex DNA (PDB: 3PIH) [19], with α-helices depicted as cylinders and β-strands as arrows. One protomer is colored by domains, whereas the other is shown in gray. The Zn atoms are shown in light green; the bound ADP molecules are shown as space-filling models. The dimer was generated by application of a crystallographic two-fold axis to the contents of the crystallographic asymmetric unit, which contains one molecule of UvrA and one strand of DNA. The location of each protein domain is projected onto the primary sequence of *Tma*UvrA, shown as a bar. (**B**) Structure of *Bacillus caldotenax (Bca)* UvrB tethered to duplex DNA via disulfide crosslinking (PDB: 6O8E) [47]. The protein is colored by domains; the bound ADP molecule is shown using space-filling model. The location of each protein domain is projected onto the primary sequence of *Bca*UvrB, shown as a bar. (**C**) Structures of *Thermotoga maritima (Tma)* UvrC catalytic domains and DNA-binding domain. The GIY-YIG catalytic domain for 3′ incision (PDB: 1Y1D) [48] and the RNase H-like catalytic domain for 5′ incision (PDB: 2NRZ) [49] are shown in cyan and yellow, respectively. Amino acid residues that have been identified as important for activity are shown as sticks; the bound divalent metal ions and the coordinating water molecules are shown as green and red spheres, respectively. The (HhH)_2_ DNA binding domain is shown in light red. The location of each domain is projected onto the primary sequence of *Tma*UvrC, shown as a bar. Dashed regions represent parts of UvrC whose structures are not currently available. (**D**) Structure of *Escherichia coli (Eco)* Mfd (PDB: 2EYQ) [50]. The protein is colored by domains. The location of each protein domain is projected onto the primary sequence of *Eco*Mfd, shown as a bar.

During GGR, the UvrA protein operates as the first responder to lesions in the genome. UvrA is a tandem ABC (ATP binding cassette) ATPase [31,32] with a dimeric molecular weight of over 200 kDa. ABC proteins comprise a large family that use the energy of ATP binding and hydrolysis to perform work in the cell, most of which is associated with transport across cellular membranes. UvrA is, however, one of the few ABC ATPases that performs transactions with nucleic acids [33,34]. Conserved among ABC proteins is a 250-amino acid nucleotide binding domain (NBD), with a number of sequence/structural motifs, namely the Walker A/P loop, the Q loop, the ABC signature motif, the Walker B sequence, the D loop and the H loop/switch [35,36]. In a typical ABC protein, two protein chains each contribute an NBD to the complete dimeric entity. The physiological ensemble features two composite ABC-style nucleotide binding sites; the ATP binding domain (Walker A/P loop, Q loop, Walker B, D loop and H loop/switch) from one chain partners with the ABC signature (SIG) of the second and vice versa. UvrA exhibits features of a typical ABC protein but also harbors several unique elaborations. First, the two NBDs (termed NBD-I and NBD-II) that comprise a functional ABC ATPase unit are found on the same protein chain. Second, the active entity of UvrA is a dimer and thus features a total of four nucleotide binding sites. The nucleotide binding sites are designated as proximal and distal based on their relationship to the dimer interface [37]. The proximal site has been implicated in the interactions of UvrA with UvrB and DNA [21,38], whereas the distal site has been reported to play a key role in the discrimination of native from damaged DNA [21,39,40,41]. Third, the UvrA protein encompasses a number of unique elements not found in any other ABC protein, namely the UvrB binding domain, three zinc modules (Zn1, Zn2 and Zn3), a hairpin element connected to the Zn3 module (Zn3hp) and the insertion domain (ID). Among these unique elements, the Zn3hp and the insertion domain participate in contacts with DNA [19,42,43,44]. Currently, there are eight UvrA structural models from four bacterial species (PDB IDs = 2R6F, 2VF7, 2VF8, 3PIH, 3UWX, 3UX8, 3ZQJ, 6N9L). The models populate two dimeric configurations [19,21,37,44,45,46]; considerable similarity in conformation is found in the ABC NBD portion of each protein, while the unique elements occupy divergent relative positions (Figure 2A).

## 3. UvrB is a Damage Specific Helicase that Prepares DNA for Excision

UvrB is a member of the Superfamily II grouping of helicases, as indicated by presence of conserved motifs in domains 1a and 3 [51,52]. UvrB couples ATP binding and hydrolysis with domain motion to implement 5′→3′ ssDNA translocase activity [53,54], using a nucleotide binding site located at the interface between domain 1a and [47,51]. The importance of the ATPase and translocase activity is indicated by the finding that mutation of the conserved K45 residue in the Walker A motif to Ala [53,55] renders the protein incapable of forming the pre-incision complex [56]. Notably, the activity of UvrB is tightly regulated, with its ATPase and translocase activities suppressed by the presence of the auto-inhibitory domain 4 [57]; this inhibition is relieved in the presence of UvrA and ssDNA [57,58].

Unlike other helicases and despite possessing all the structural elements required for ATP-dependent DNA unwinding, UvrB only has weak strand-separating activity in the presence of UvrA. UvrB is only able to unwind ~ 20 base pairs of DNA in a strand displacement assay [54,59]. Strand separation during translocation is proposed to occur via insertion of a β-hairpin sub-structure into the duplex, with two separated strands passing on either side of the hairpin (Figure 2B). Importantly, helicase activity of UvrB is blocked by the presence of damaged DNA bases [53,54]. Interaction with damaged DNA requires several conserved hydrophobic residues at the base of the β-hairpin [47,60,61,62,63]. Until recently, it was unclear which strand, native or damaged, is mapped to which side of the hairpin [47].

## 4. UvrC is a Damage Specific Dual Nuclease

UvrC is a dual endonuclease recruited to the UvrB•DNA pre-incision complex to introduce incisions on either side of the lesion [23]. The first incision is damage-dependent and is made at the 4th or 5th phosphodiester bond 3′ to the lesion by the N-terminal domain of UvrC, while the second incision is made at the 8th phosphodiester bond 5′ to the lesion by another endonuclease domain located towards the C-terminus [52]. Following both incisions, an additional damage-independent cleavage is often observed at the 15th phosphodiester bond 5′ to the lesion; this extra incision is carried out by the same catalytic domain responsible for the damage-dependent 5′ incision [64].

Biochemical experiments have shown that both incisions are made by a single UvrC molecule within one binding event and are regulated by binding and hydrolysis of ATP in the UvrB•UvrC•DNA complex [65]. The 3′ incision is made by the N-terminal domain of UvrC, which harbors a GIY-YIG motif seen in other endonucleases [66]. Kisker and coworkers have proposed that the UvrC 3′ endonuclease domain uses a metal-ion-based mechanism to catalyze cleavage of the phosphodiester bond [48]. On the other hand, the 5′ incision is catalyzed by the C-terminal domain of UvrC. Structure-function studies [67], in combination with structural similarity to RNaseH enzymes, suggest involvement of a two-metal mechanism in the 5′ incision [49].

Although the complete structure of UvrC remains to be described, several additional structural features are notable. The first is an element (residues 191–233) that resides between the two endonuclease catalytic domains of UvrC (Figure 2C) and exhibits sequence similarity to the C-terminal domain of UvrB; this element is required for the interaction with UvrB. Although the structure of this UvrC element is unknown, a structure is available for the homologous element from UvrB [68,69]. The arrangement in the crystal features two copies of UvrB C-terminal domain in a head-to-head dimeric configuration, with dimerization mediated by hydrophobic interactions and salt bridges between highly conserved residues located within and around the loop region. This crystallographic interaction provided a basis for constructing a model of contacts made by UvrB and UvrC in the complex; this model is supported by mutational analyses. Notably, interaction between these two segments on UvrB and UvrC is required to form the pre-incision complex and complete the 3′ incision [70], as well as the additional damage-independent 5′ incision [64].

The other structural feature of UvrC is the DNA-binding domain comprising two helix-hairpin-helix motifs (HhH)_2_ that is located at the C-terminus of UvrC [71] (Figure 2C). This domain is required for binding to ssDNA, the 5′ incision and the additional damage-independent incision at the 15th bond 5′ to the lesion [64,72]. In addition, it may also play a role in the 3′ incision in certain sequence contexts [73]. The (HhH)_2_ DNA-binding domain is observed to adopt significantly different positions relative to the C-terminal 5′ catalytic domain of UvrC [49]. Intrinsic conformational flexibility between the 5′ endonuclease domain and the other domains of UvrC could be important for its ability to incise DNA substrates containing different lesions by allowing UvrC to adjust its positioning of the active site for 5′ incision. Based on the structural similarity [74], a model was proposed in which UvrC uses the conserved GφG motif, where φ is a hydrophobic residue, in the (HhH)_2_ domain to interact with DNA. The relationship between the two nuclease domains and the structural elements noted above during NER will require additional structural and biochemical analyses to elucidate.

## 5. Mfd Structure and Function

Mfd is a multi-domain protein that undergoes a large conformational change regulated by ATP binding and hydrolysis during its functional cycle [24]. Composed of eight domains (D1a, D1b, D2, D3, D4, D5, D6 and D7), Mfd is organized around four distinct functional regions as follows (Figure 2D). The UvrB homology module is located at the N-terminus of Mfd and is made up of domains D1a, D1b and D2). The RNA polymerase interacting domain (RID) consists of domain D4. Domains D5 and D6 together constitute the translocase module, whereas domain D7 is responsible for auto-inhibition via interaction with domain D2. These functional regions enable Mfd to play role in the removal of stalled RNAP, as well as the recruitment of the NER apparatus.

The N-terminal UvrB homology module (D1a, D1b and D2) exhibits structural and sequence similarity to domains 1a, 1b and 2 of UvrB [50]. Upon DNA binding, the UvrB homology module was observed to undergo a large swivel motion compared to its position in apo-Mfd; this structural change has been proposed to link the recruitment of UvrA to the dissociation of Mfd from DNA [75]. In place of the highly conserved β-hairpin that has been shown to be important for damage binding in UvrB, Mfd harbors a short loop. In addition, the UvrB homology module of Mfd lacks ATPase motifs [76]. As a result, the UvrB homology module of Mfd lacks ATPase activity and is incapable of DNA unwinding and binding to DNA lesions [76]. Within the UvrB homology module, domain D2 provides the binding surface for UvrA [26] and is the most highly conserved region between Mfd and UvrB.

Domain D4, the RNAP interaction domain (RID), has structural similarity to the Tudor-like domain, a part of bacterial elongation factor that interacts with the β-subunit of the RNAP [50]. RID is not unique to Mfd; it is also found in a family of CarD transcription factors. In both Mfd and CarD, the RID is used to interact with subunit β1 of RNAP [24].

Domains D5 and D6 of Mfd are homologous to RecG, the bacterial motor protein that couples ATP hydrolysis to double-stranded DNA translocation. However, unlike other helicases, domain D5 and D6 of Mfd cannot separate the DNA strands [77]. In RecG, the crucial structural element TRG (translocation in RecG) motif couples ATP hydrolysis with rigid-body motion of two translocase domains for translocation on the DNA strands [78,79]. In Mfd, the TRG motif is located at the end of domain 6. Substitution of key residues in the TRG motif of Mfd impaired the ability of Mfd to displace RNAP, suggesting that the TRG motif is essential for the displacement of RNAP [80]. A structure of Mfd in complex with DNA also revealed that TRG is involved in Mfd translocation [75], supporting the above hypothesis.

Domain D7, located at the C-terminus of Mfd, is an auto-inhibitory domain. Domain D7 interacts with domain D2 and occludes the UvrA binding interface in apo-Mfd [50,76]. Domain D7 undergoes a large conformational change in the presence of ATP, resulting in the extended Mfd structure with exposed UvrA binding surface [26].

## 6. Global Genome Repair (GGR)

### 6.1. Discrimination of Native DNA from Damaged by UvrA

Of all the DNA repair pathways, NER is distinguished by its capacity to process widely varying DNA lesions [6,52], including those processed by mechanisms specific to particular lesions [52]. This feature of NER raises a key unresolved question: how do a series of highly conserved proteins accurately identify and repair a diverse set of DNA lesions. It is axiomatic that the structurally and chemically diverse nature of lesions processed by NER precludes direct recognition of DNA adducts. Rather, NER proteins likely recognize common abnormalities of the DNA double helix induced by the structurally varying lesions. Prior studies have suggested that properties such as reduced stability of the double helix, state of unwinding [81,82], structural distortions, changes in electrostatic properties [83,84], alterations in base stacking or local flexibility [85], as well as changes in DNA conformational dynamics [86,87] could be factors leveraged by UvrA to distinguish damaged from native DNA. Decisively addressing this question for both bacterial and eukaryotic NER remains a major line of investigation.

UvrA uses the energy from ATP binding and hydrolysis (below) to detect widely varying damaged DNA among the vast excess of normal DNA in the genome. To perform this important function, UvrA must have the capacity to discriminate native DNA from a series of chemically and structurally distinct lesions, including diverse adducts, mismatches, nicks and gaps [52,88,89]. UvrA exhibits a 2–5 fold preference for damaged DNA in comparison to undamaged DNA [42,90]. While precise values for specificity of NER in vivo are difficult to establish owing to multiple confounding factors [91,92], it is clear that small differences in binding affinity alone cannot account for the exquisite specificity of NER, which is assumed to repair the bacterial genome within the cell division cycle [7]. It has been suggested that kinetic proofreading may play a key role in supplementing differences in binding affinity to explain precision of NER [30,93,94,95,96]. Kinetic proofreading is a mechanism that allows biological reactions to achieve higher specificity beyond that afforded by differences in free energies of binding between correct and incorrect substrates [97]. The enhanced specificity is made possible by unidirectional energy-utilizing intermediate steps, which generate delays between initial target binding and catalysis; the correct substrate complex survives these delays and moves to the next step, while incorrect substrates do not and dissociate. During NER, the UvrA and UvrB proteins use the energy of ATP binding and hydrolysis to implement a precise sequence of events that begins with damage detection by UvrA and ends with UvrB at the lesion, poised to recruit the UvrC nuclease.

The molecular dance performed by UvrA and UvrB on DNA has been considerably illuminated by insightful biochemical, structural and single-molecules studies. The physiological DNA damage sensor in GGR is a complex between UvrA and UvrB whose in vitro stoichiometry has been variously estimated as UvrA_2_B_2_ or UvrA_2_B_1_ [21,98,99,100,101,102]. The UvrA_2_B_2_ complex uses three different search modes to locate damaged DNA: (1) free diffusion, in which the complex randomly changes position on DNA; (2) directed motion, where the complex migrates with directionality on DNA; and 3) paused motion, which is characterized by long pauses followed by short bursts of movement [94,98]. On the other hand, a recent in vivo single-molecule analysis suggested that UvrA alone performs the search for a lesion and that UvrB is subsequently recruited to the damage site by UvrA [38]. With either model, UvrA plays the key role in damage recognition as it is the first protein that establishes contact with the DNA before handing it off to UvrB [18].

Although the main contours of damage discrimination during NER have been described, there are indications that either the standard model is incomplete or that there are species-specific mechanisms. The finding that alkylated DNA damage, which is ordinarily repaired by direct reversal [103,104] or base excision repair [105], can also be repaired by NER with the help of alkyltransferase-like proteins (ATLs) [106], suggests that collaborations with other proteins may be a little understood feature of UvrA. Studies show that the ATL protein contacts both UvrA and UvrC [107]. In addition, UvrA binds to oligomeric ATL on both damaged and undamaged DNA and the resulting complex translocates on the DNA duplex [108]. The finding that *E. coli* and *Bacillus caldotenax* UvrB•UvrC complex can perform NER in the absence of UvrA [109], that *Mycobacterium tuberculosis* UvrA and UvrC may directly interact [110] and that UvrA, UvrB and UvrC can form a complex termed the repairosome [109,111], each challenges the standard model; these observarions imply that additional chapters of the NER story remain to be uncovered.

The precise molecular signature in damaged DNA that enables its discrimination from native DNA, along with structural changes experienced by UvrA during GGR, remains to be described. However, structural and biochemical analyses have primed several hypotheses that provide a framework for understanding both features [19,20,21,22]. The crystal structure of UvrA bound to a symmetric DNA duplex with a lesion on both strands suggested indirect readout of the presence of damage. Inspection of the structure, along with structure-function studies lay the foundation for understanding two properties of the UvrA•DNA complex: non-specific interactions that would be required for genome scanning and structural changes associated with formation of a tight complex on damaged DNA. The signature domain II and the insertion domain contribute the majority of contacts to DNA [19,21], as anticipated [37,43]. In addition, it is now clear that one locus of damage-specific contacts is the β-hairpin/Zn-binding module (Zn3hp) element; inserted into the ABC signature domain II. This structural element is unique to UvrA and is coupled to nucleotide dynamics at the proximal site [41]. The position of this element is altered upon binding DNA [19]. Furthermore, compromising the integrity of this element by deletion of the Zn-binding module [42,43,109] or restricting its motion by cross-linking [41] impairs the capacity to discriminate damaged from native DNA.

The data above have led to two proposed frameworks for understanding discrimination between native and damaged DNA by UvrA. The first suggests that UvrA may subject DNA to a ‘stress test’ by coupling ATP binding and hydrolysis to unwinding, stretching and compressing DNA [22]. Damaged DNA, which is known to harbor non-native base stacking [85], is more likely to bend and buckle, leading to strand separation [85]; damaged DNA fails this test and is passed to the damage specific steps of NER [22]. A second model arose from study of the two dimer configurations (‘open tray’ and ‘closed groove’, below) of UvrA; it was suggested that these two configurations subject DNA to a shape test: natively shaped DNA passes the test, while aberrantly shaped lesion-containing DNA fails and is shunted towards the damage specific stages of NER [21]. Notably, the two hypotheses are not mutually exclusive and the actual mechanism may blend elements of both models.

### 6.2. UvrA Mediates a Match between Damaged DNA and UvrB

UvrA is not only the first responder to the presence of damaged DNA but also the molecular matchmaker that enables delivery of UvrB to the precise site of the lesion. Molecular matchmakers are proposed to be special catalysts that apply ATP binding and hydrolysis on a pair of molecules to enable conformations competent to bind each other; absent the matchmaker, interactions between this pair of molecules is not favorable [112]. In this scheme, UvrA make a match between UvrB and damaged DNA. The process of discrimination and subsequent tight binding of damaged DNA represents one half of the match, while binding of UvrB represents the second half. The order of binding to the matchmaker is not constrained; either damaged DNA or UvrB may be bound by UvrA first. Atomic structures of both halves of the match: UvrA bound to damaged DNA [19] and UvrA bound to UvrB [21] have been described. The configuration of the UvrA_2_B_2_-complex on DNA, however, remains to be visualized.

**Figure 3:**
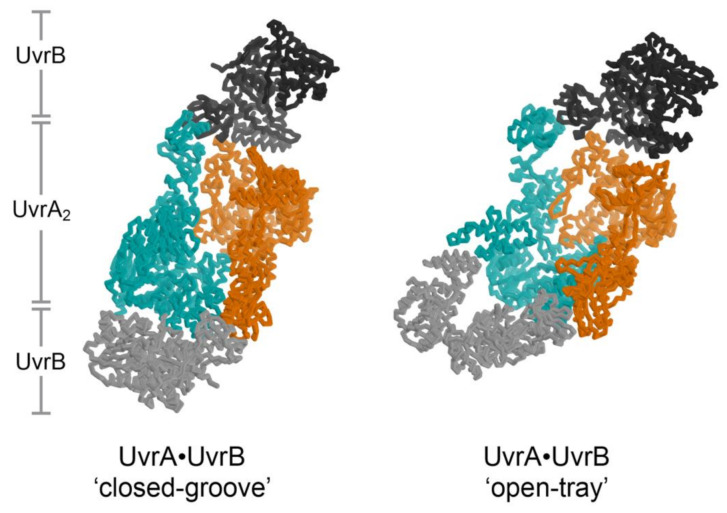
Two models for the UvrA_2_B_2_ complex. The two UvrA protomers are shown in cyan and orange, while the two UvrB molecules are shown in different shades of gray. The crystal structure of the UvrA_2_B_2_ complex has the UvrA dimer in the ‘closed-grooved’ configuration [22] (**left**), whereas the negative stained electron microscopy structure of UvrA_2_B_2_ complex in the presence of DNA has the UvrA dimer in the ‘open-tray’ configuration (**right**) [21]. Although dsDNA was included in the EM sample, the DNA is not visible in the electron density map and is not included in the model. The two UvrA_2_B_2_ models are aligned on the orange UvrA protomer. With this alignment the cyan UvrA protomers in the two models are related by a 50° helical rotation (50° rotation, 20 Å translation) about the axis approximately parallel to the presumed DNA binding path.

The structure of the UvrA_2_B_2_ complex [21] unexpectedly revealed a dimeric arrangement of UvrA that is distinct from that observed in every structure of isolated UvrA [19,37,44,45,46]. An important consequence of this distinct configuration is the alteration of the UvrA surface that binds DNA from a relatively open shape (termed the ‘open tray’ dimer) compatible with the widely varying conformations of damaged DNA to a ‘closed groove’ shape that appears solely compatible with native DNA (Figure 3, left). Damage discrimination was suggested to feature detection of shape differences between native and lesion-containing DNA. UvrA was also found to bind to two UvrB molecules at the periphery of the ensemble, far from the expected position of the lesion. Recent analysis of the UvrA_2_B_2_ complex using negative stain electron microscopy at 25 Å resolution confirmed the UvrB-UvrA-UvrA-UvrB disposition of subunits. Computational fitting studies with existing UvrA and UvrB models led the authors to conclude that UvrA, within the complex, adopted the ‘open tray’ dimeric form [20] (Figure 3, right). This is an intriguing finding that lends support to the idea that UvrA may interconvert between two dimeric states during NER but also one that will require higher resolution studies to more firmly establish. Additional structural studies will be required to further reveal conformation switching by UvrA within its complex with UvrB and whether the DNA is wrapped around UvrB [113,114] within the complex.

### 6.3. Dissociation of UvrA from DNA and Formation of the Pre-Incision Complex

The dissociation of UvrA from the damage-sensing complex, the orchestration of the initial loading of UvrB and subsequent translocation to the lesion using its 5′→3′ ssDNA translocation activity, the number of UvrB molecules loaded by UvrA and the identity of the DNA strand presented to UvrC have been enduring mysteries of the NER pathway. It has long been known that dissociation of UvrA from the damage sensing complex is a pre-requisite for loading of UvrB at the lesion to form the pre-incision complex [100], however, the mechanisms that underlie this process are incompletely understood. Prior studies have established that ATP hydrolysis by UvrA leads to its dissociation from the DNA [115] and that ATP binding and hydrolysis accompany translocation of UvrB towards the lesion [53,54]. The possibility that UvrA could load two UvrB molecules on distinct strands, approximately 80 Å away on either side of the lesion [21], provides a structural framework for understanding how the UvrA_2_B_2_•damaged DNA complex transitions to the UvrB•lesion pre-incision complex. This transition has been envisioned via two models: the conformational change and the translocation models [21]. In the first model, a large conformational change in the UvrA_2_B_2_ complex delivers UvrB from its peripheral location in the complex to its final location at the lesion site; precise positioning at the lesion is enabled by helicase activity of UvrB, which accompanies dissociation of UvrA. By contrast, the translocation model implies that UvrA dissociates prior to translocation of UvrB to the site of the lesion [20,21]. In either model, loading two UvrB molecules on distinct strands could serve as a mechanism to identify the lesion-containing strand for presentation to the UvrC nuclease.

The crystal structure of the UvrA_2_B_2_ complex provided a structural basis for understanding how ATP hydrolysis at the UvrA proximal site and conformation switching by the signature domain II and possibly the UvrB-binding domain, enables dissociation [21]. A recent single molecule live cell imaging study showed that in the absence of UvrB, UvrA displays a lifetime of ~20–30 s on damaged DNA; a short-lived complex (~1.4 s) was also observed but not extensively analyzed. The presence of UvrB reduced the lifetime of the long-lived population to ~8.7 s, whereas the lifetime of the short-lived population remained unchanged. These measurements capture the dynamics of UvrA loading UvrB onto lesion DNA prior to its dissociation [27]. Notably, a prior single molecule study found no evidence that UvrB reduces the lifetime of UvrA on DNA in vitro [98].

The finding that UvrB may approach the lesion from the 5’ side [116] has been more precisely defined by recent structural, crosslinking and biochemical studies. Two orthogonal experiments directly documented translocation. First, Lee et al. crosslinked UvrB to DNA on the 5′ side of the lesion; release of the crosslink exposed the 5′→3′ translocation activity, which led to stalling of UvrB at the lesion [47]. Second, Jaciuk et al. used substrates with two lesions, variously spaced, to show that only the lesion closest to the 5′ end was incised; this finding implies that the single processed lesion stalls translocation by UvrB [20]. Although both studies confirm that UvrB translocates in the 5′→3′ direction and is stalled by the lesion during NER, they do not reveal the initial site of UvrB binding relative to the lesion.

Although considerable evidence has been developed to support the idea that two UvrB molecules are loaded by UvrA [99,101,102], possibly on distinct strands [21], this finding must be reconciled with experiments that show that a single UvrB molecule is bound to the UvrC nuclease in the incision complex [101]. An important question associated with the transition from two to one UvrB molecules in the incision complex centers on which DNA strand, damaged or undamaged, is bound under the β-hairpin of UvrB. Two independent studies definitively showed that it is the damaged strand that is under the β-hairpin of UvrB for presentation to the UvrC nuclease [20,47]. Furthermore, a crystal structure of UvrB bound to mismatch containing DNA lays the foundation for understanding the precise geometry of the cuts made by the dual nuclease UvrC, eight positions 5’ of the lesion and five positions 3’ of the lesion [47].

### 6.4. Two Types of ABC ATPase Sites on UvrA Power Damage Detection and UvrB Binding

It has been known for nearly 35 years that ATP is involved in steps of NER mediated by UvrA and UvrB [16,39,40,56,58,65,99,117,118,119,120], however, precise roles for the nucleotide sites have yet to be decisively established. Prior studies have suggested roles for ATP in stability of the UvrA dimer [119,121,122], interaction with UvrB [100] and damage discrimination [16,39,40,41,46,118,120], with strong evidence for mixed nucleotide states and cooperativity between sites [40,46,118,120,122]. Structural and biochemical evidence implicated nucleotide dynamics at the proximal site in interactions with UvrB and DNA [21,38], while discrimination of native from damaged DNA was hypothesized to involve the distal site [21,39,40,41]. The finding that UvrA may tune its activity depending on the lesion [46], that NER efficiency is dependent on sequence context [123,124] and that bulky DNA lesions can be repaired in the absence of ATP hydrolysis by UvrA [120] is intriguing and requires further exploration. The extensive structural database of other ABC-family proteins [36,125] provide a basis for predicting or understanding conformation-switching in UvrA during NER. However, the presence of two types of ATP binding sites in UvrA, which could each transition through three nucleotide states (empty, ADP and ATP) and are coupled, giving rise to eight possible nucleotide combinations [46], underscores challenges in understanding the role of ATP during NER.

**Figure 4:**
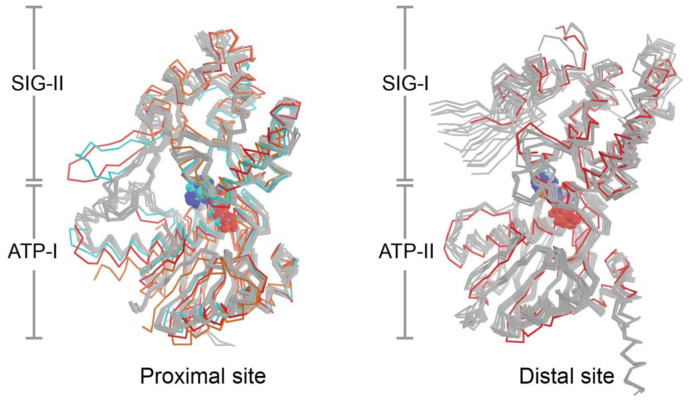
The proximal and distal nucleotide binding sites of UvrA. The nucleotide-binding domains that make up the proximal and distal nucleotide-binding sites from Protein Data Bank (PDB) entries 2R6F [37], 3UX8 [21], 3UWX [21], 3PIH [19], 3ZQJ [45], 2VF7 [44], 2VF8 [44] and 6N9L [46] were superimposed on the ATP-binding (ATP) domain. The structures are colored in gray save for 3UX8 (orange), 3PIH (red) and 6N9L (cyan) in the proximal site alignment; and 3PIH (red) in the distal site alignment. The colored structures deviate from remaining members of the group by rotation of their respective signature (SIG) domain.

The current set of eight crystal structures from four bacterial species provide 24 independent views of the NBDs and composite nucleotide-binding sites of UvrA, however, the structural configuration formed in the presence of ATP is currently lacking. This limits efforts to understand how nucleotide binding and hydrolysis are coupled to DNA damage sensing. Superposition of all available nucleotide binding site structures on the ATP binding domains (Figure 4) reveals a low RMSD (proximal: 1.19 Å, distal: 1.06 Å). With the exception of the conformers that lack nucleotide or are bound to dsDNA, the ABC signature domains are found to populate a narrow range of configurations that are within 1–10° (average proximal: 4.7°; average distal; 7.6°) of the reference molecule (PDB entry 2R6F); the corresponding values for sites that lacked nucleotide or are bound to DNA are in the 18–20° range (Figure 4). Two lines of evidence indicate that UvrA likely undergoes substantial conformational changes during NER. First, restricting dynamics of the β-hairpin in the Zn3 module (Zn3hp) of UvrA using disulfide cross-linking disrupts its ATPase activity and capacity to load UvrB onto damaged DNA; this dynamism requires a functional distal nucleotide binding site [41]. Second, the NBDs of ABC ATPases in membrane transporters, as exemplified by the TAP transporter [125], have been observed to exhibit significantly larger angular and distance changes during the functional cycle than those currently seen with the current set of UvrA structures. The fact that the nucleotide binding sites of most the available UvrA structures are filled with ADP and the finding that the motion of domains most expected to switch conformation is restricted by crystal contacts limits structural insights into the UvrA ATP cycle during NER.

Biochemical and biological studies have, however, provided new and important clues into nucleotide dynamics of UvrA during NER. Detailed analysis of pre-steady state kinetics revealed that UvrA programs differential responses by the proximal and distal ATPase sites to absence of DNA or presence of either native or damaged DNA [46]. The two types of sites were found to bind ATP with a considerable difference in affinity [21,40,46]. The proximal (P) site bound nucleotide weakly, while the distal (D) site bound nucleotide tightly; this finding could be correlated to the subtle difference in amino acids surrounding the two types of nucleotide binding sites. In the absence of DNA, the weak proximal site hydrolyzes ATP rapidly, which in turn stimulates turnover at the tight distal site to give rise to a P_2ATP_-D_2ATP_ species. Interestingly, provision of native or damaged DNA provokes divergent responses: a P_2ATP_-D_2ATP_ species accumulates when native DNA is detected, in contrast, damaged DNA leads UvrA to populate a P_2ATP_-D_empty_ or P_2ATP_-D_ATP_ states [46]. This remarkable repertoire of varied responses likely set the stage for transitioning towards to the damage specific stages of NER.

Insights into roles played by the ATPase activities of UvrA have also emerged from thoughtful experiments on NER in live *E. coli* [38]. This study showed that UvrA requires ATP binding at both proximal and distal sites to load UvrB. Closer analysis revealed that ablation of ATP binding at the proximal site (Walker A, K37A) leaves the capacity of UvrA to locate lesions relatively untouched in comparison to wild-type. Further, impeding ATP hydrolysis by the proximal site (Walker B, E514A) results in a species that binds tightly to DNA regardless of the presence of the damage; this mutant was also deficient in loading UvrB onto damaged DNA. Disruption of the distal site (Walker A: K646A) revealed a crucial role for the distal site in finding the lesion. Impairing the capacity of the distal site to hydrolyze nucleotide turned UvrA into a tight DNA binding protein, similar to what was measured with the proximal ATP hydrolytic mutant. In contrast to the proximal site, disruption of ATP hydrolysis at the distal site left the UvrB-loading capacity of UvrA unscathed. Further, the authors infer that UvrB loading requires a functional proximal site but only the capacity of the distal site to bind ATP.

## 7. Transcription-Coupled Repair (TCR): RNA Polymerase as the DNA Damage Sensor

The first evidence of transcription coupled repair (TCR) was reported decades ago [126,127,128,129]. Studies in both mammalian cells and *E. coli* showed that actively transcribed genes were repaired much more rapidly than the rest of the genome and that this could be attributed to the repair of the ‘template’ strand [127,128,129]. Subsequently, it was revealed that the strand-specific repair is due to a specialized form of NER and that the protein responsible for the coupling of excision repair with transcription, the transcription-repair coupling factor (TRCF), in bacteria is Mfd (mutation frequency decline) [130,131]. The Mfd protein increases the rate and the efficiency of the repair, compared to GGR [132,133,134]. In addition, the efficiency of repair is significantly increased for substrates not effectively recognized by UvrA, such as cyclobutane pyrimidine dimer (CPD) [135].

The transcription coupled repair pathway operates when a bulky DNA lesion on the template strand causes stalling of the transcription elongation complex (EC); the lesion inhibits transcription as well as hinders movement of DNA replication forks. During TCR, the stalled RNAP and the nascent transcript are removed from the DNA template and the UvrA or UvrA_2_B_2_ complex is loaded onto the DNA to initiate excision repair of the DNA lesion. According to the ‘pervasive transcription’ model [136], the entire genome is being transcribed, albeit at low levels. In this view, RNAP is a particularly useful DNA damage sensor in that it is persistently surveilling the genome for lesions using efficient one-dimensional diffusion.

Since its discovery, TCR was believed to be mediated by the Mfd protein [77,130,131,133,137], which recognizes the stalled RNAP and displaces it by forward translocation [25]. However, recent studies suggest that there could be an alternative TCR pathway that is independent of Mfd and involves RNAP backtracking elicited by UvrD [28,29,138]. Both of these pathways are discussed below.

### 7.1. Mfd-Dependent TCR

TCR-Mfd is triggered when the elongating RNAP stalls at a lesion, however, stalling obscures the damaged DNA segment from processing by repair proteins. To repair such a lesion, the stalled RNAP must first be displaced. Mfd binds to the stalled RNAP immediately upstream of the arrest site and displaces the stalled RNAP from the DNA strand by pushing it forward using an ATP-dependent DNA translocase activity, orchestrated by domains 5 and 6 [25,75]. Single-molecule fluorescence imaging analysis revealed that as the RNAP is evicted from the DNA, the RNA is also released [139]. However, the displaced RNAP still associated with the slowly translocating Mfd [139]. Notably, Mfd appears to fulfill additional roles that do not involve presence of lesions, since its association with RNAP takes place absent DNA damage [140]. Further, a recent single molecule study showed that Mfd translocates on genomic DNA autonomously and remediates RNAP stalled at pause sites, in the absence of lesions [141,142]. Lastly, roles for Mfd beyond functioning in a damaged DNA context is highlighted by an activity that rescues RNAPs whose forward progress is inhibited by protein blocks on DNA [143,144]. Roles played by Mfd outside of a lesion-DNA context will not be further discussed here.

The mechanisms that enable translocation by Mfd to displace RNAP remain to be clarified and likely involve a number of distinct structural intermediates in both Mfd and RNAP. It has been proposed that translocation of Mfd in the presence of bound RNAP could impose torque on the upstream DNA, causing rewinding of the upstream edge of the transcription bubble and collapse of the elongation complex [24]. The translocation activity of Mfd is regulated by the autoinhibitory domain D7 and domains D1-D3 [145,146]. Binding of Mfd to RNAP via interaction between RID and the β subunit of RNAP results in the relief of auto-inhibitory interaction and activation of ATPase and translocase activity.

To complete the repair process, Mfd recruits the core NER machinery. Isolated Mfd does not bind to UvrA, since the D2 UvrA-binding surface is masked owing to an interaction with the D7 element [50]. Interaction of Mfd with the stalled RNAP unmasks the UvrA binding element, leading to an interaction only when a lesion is encountered [26]. Engagement of UvrA to the Mfd-RNAP complex is mediated by ATP binding and hydrolysis by both Mfd [26] and the distal ATP-binding site of UvrA [27]. UvrA and UvrB together have been shown to facilitate displacement of Mfd from DNA [140,147]. Importantly, UvrB loading is required for the removal of Mfd from the DNA. This is evident by the fact that Mfd-UvrA_2_-UvrB complex formed by UvrB that is deficient in loading are impaired in the handoff of damaged DNA from Mfd to UvrA_2_B_2_ [140]. Once UvrB is loaded onto the site of the damage and the ‘pre-incision complex’ is formed, the repair reaction proceeds identically to those seen in the latter steps of GGR.

### 7.2. Does UvrD Mediate an Alternative TCR Pathway?

UvrD has recently emerged as a potential key player in TCR [29]. Although UvrD also participates in the removal of excised damaged oligonucleotide, a feature common to both GGR and TCR, it was observed that cells lacking UvrD could still support GGR but were defective in TCR [148], suggesting that the major role of UvrD is in TCR. UvrD has been reported to mediate RNAP backtracking at the site of UV lesions, which facilitates the repair of the lesion by the core NER machinery [28]. We note, however, that it has not been explicitly shown that UvrD-mediated RNAP backtracking results in the preferential repair of the transcribing strand.

During the proposed UvrD-mediated TCR, the stalled RNAP is pulled backwards to expose the lesion so that is can be detected and repaired by UvrABC [138]. Although the recruitment of UvrA_2_UvrB_2_ complex to the lesion by UvrD has not been directly documented, it has been suggested in the study by Nudler lab [28], based on the observed physical interaction between UvrD and UvrB [149,150] and between NusA and UvrA [151]. In contrast to Mfd-mediated repair, which causes transcription termination and release of the nascent transcript, the backtracking of RNAP and repair mediated by UvrD can occur without causing termination [28].

UvrD belongs to the SF1 superfamily of helicases and translocates on ssDNA with a 3′→5′ directionality [152,153]. Monomeric UvrD can translocate on DNA, however, only the dimeric UvrD has helicase activity [154]. UvrD is a highly abundant protein, with copy number comparable to RNAP [24] and it form a stable complex with RNAP [28]. Based on these observations, it has been proposed that RNAP might be “pre-loaded” with UvrD, even before the lesion is encountered [24]. UvrD uses its C-terminal domain [155] to interact with the β and β′ subunits of RNAP and binds to the upstream edge of the transcription bubble [28]. Notably, although UvrD and Mfd appear to bind to non-overlapping surface of RNAP, they cannot engage RNAP simultaneously [155]. Upon encounter of DNA damage, levels of UvrD rise as part of the SOS response [156,157]. This could promote dimerization of UvrD on RNAP, resulting in polymerase backtracking and TCR.

UvrD-mediated RNAP backtracking is assisted by the transcription elongation factor NusA and ppGpp. NusA binds to the flap region of the β subunit of RNAP [158,159,160] as it leaves the promoter region and could compete with Mfd for binding to RNAP [161]. ppGpp is a bacterial ‘alarmone’ whose cellular concentration increases during SOS response [162]. Binding of ppGpp to RNAP causes it to adopt a conformation that is prone to backtracking [163]. Taken together, these interactions could facilitate the alternative UvrD-mediated TCR pathway [29].

## 8. Future Directions

Recent structural, biochemical, genetic and single molecule approaches have provided evidence that support and expand our understanding of the mechanisms that underlie bacterial NER. These studies have shed new light on mechanisms deployed by UvrA and UvrB during lesion recognition. As well, insights have emerged at the interface between UvrA and transcription as part of transcription-coupled repair. A number of questions, however, are still left incompletely understood. What are the precise structural changes in UvrA that accompany binding to damaged DNA? What are the precise role(s) for the ATP sites on UvrA and UvrB play during NER? Does UvrA subject DNA to ATP-hydrolysis driven stress or shape tests? Or is the damage verification performed by UvrB the critical event in damage DNA discrimination? How do the two UvrB molecules that could be loaded by UvrA make their way to the lesion? How is the UvrC endonuclease activated upon entry to the UvrB-damaged DNA complex? What is the architecture of the UvrA-UvrB-UvrC repairosome? How does UvrA orchestrate UvrB loading while interfacing with the Mfd protein during transcription coupled repair? Does UvrA’s interaction with alkyltransferase-like proteins imply that other chapters in the UvrA-UvrB-UvrC story remain to be written?
